# The Relationship between the Need for Closure and Coronavirus Fear: The Mediating Effect of Beliefs in Conspiracy Theories about COVID-19

**DOI:** 10.3390/ijerph192214789

**Published:** 2022-11-10

**Authors:** Sara Staszak, Julia Maciejowska, Wiktoria Urjasz, Tomasz Misiuro, Andrzej Cudo

**Affiliations:** 1 Institute of Psychology, University of Zielona Góra, 65-417 Zielona Góra, Poland; 2 Department of Experimental Psychology, The John Paul II Catholic University of Lublin, 20-950 Lublin, Poland

**Keywords:** conspiracy theories, conspiracy beliefs, coronavirus fear, the need for cognitive closure, COVID-19

## Abstract

This study investigated the relationship between fear of the coronavirus, belief in COVID-19 conspiracy theories, and dimensions of the need for cognitive closure. As there is evidence of associations between these variables, we hypothesized that the relationship between the need for closure dimensions and coronavirus fear may be mediated by conspiracy beliefs about COVID-19. We analyzed the results from 380 individuals who completed online versions of three scales: the Fear of COVID-19 Scale, a short version of the Need for Closure Scale, and—designed for this study—the Conspiracy Theories about the Coronavirus Scale. The results showed that belief in COVID-19 conspiracy theories fully mediated the relationship between the fear of the coronavirus and avoidance of ambiguity, as well as closed-mindedness. The findings provided evidence that beliefs in conspiracy theories may play a significant role in reducing the level of coronavirus fear in people with high levels of these traits. In addition, a partial mediation between the fear of the coronavirus and the need for predictability was found. The limitations and implications of the research are discussed.

## 1. Introduction

A novel coronavirus, SARS-CoV-2, was identified in China in late 2019. Due to the rapid increase in the number of cases, the World Health Organization announced the COVID-19 global pandemic in March 2020 [1]. Along with reliable information about the spread of the coronavirus, appropriate self-isolation times, and the number of COVID-19 cases, a considerable amount of inaccurate and unreliable information appeared in the media on a number of issues, including the possible origins of the coronavirus, treatment methods, and the severity of the disease. Large numbers of fake news articles appeared online, including false and/or misleading information about the origin of SARS-CoV-2, home remedies and “cures” for it, and ways of preventing catching the virus. Simultaneously, there was an increase in conspiracy theories suggesting that official sources of information deliberately overestimated the harmfulness of the disease, as well as irrational beliefs pointing to the connection of the outbreak with the 5G network, or that COVID-19 was accurately predicted and even caused by Nostradamus, Bill Gates, or the Simpsons [2,3]. There are also theories suggesting that vaccines against the virus (or medicine to help mitigate its effects) have been available for a long time (certainly dating from the original outbreak), but that these are exclusively available to the elite [4].

These misconceptions can have severe health or social consequences for those who believe in them. Considering the diversity and commonness of COVID-19 conspiracy theories, it is important to analyze the drivers of these beliefs. In this context, the key question is how individuals search for and analyze the information about the coronavirus, and how this information relates to their mental health. Possible factors that may explain the emergence and development of COVID-19 conspiracy theories include the need for cognitive closure as an epistemic motivation to search for and analyze incoming information and the fear of the coronavirus as a negative mental state associated with the coronavirus pandemic. Therefore, the aim of this research was to determine the relationship between these factors and the strength of beliefs in conspiracy theories about COVID-19.

### 1.1. Coronavirus Fear

Infectious diseases, such as COVID-19, are considered to be more frightening than other diseases [5,6]. This may be related to their mortality, morbidity, and transmission rates, as well as to the invisibility of pathogens that transmit them. Recent research shows that stress and anxiety levels increased during the coronavirus pandemic [7,8]. The prevalence of mostly negative news coverage in the media regarding the coronavirus could contribute to fear and concerns about this situation [9].

Schimmenti et al. [10] identified four dimensions of the fear of the coronavirus: (1) the fear of the body/fear for the body; (2) the fear of significant others/fear for significant others; (3) the fear of not knowing/fear of knowing; and (4) the fear of taking action/fear of inaction. Previous research showed that fear of the coronavirus was positively correlated to general anxiety, worry, intolerance of uncertainty, and risk for loved ones, but negatively correlated to overall health, the meaning of life, and alternative ways of thinking [11,12]. Furthermore, knowing someone diagnosed with COVID-19, loneliness, death obsession, and preoccupation with God can be considered to be predictors of fear of the coronavirus [13]. Much of the research has focused on the sociopsychological predictors of coronavirus fear. However, factors related to the perception of information about the coronavirus also change the level of that fear [14,15,16].

In the context of a decreasing and/or diminished interest in or avoidance of ambiguity, news about COVID-19 was found to be associated with younger ages, greater post-traumatic stress symptoms, decreasing fear of COVID-19, and less frequent use of healthcare professionals in accessing COVID-19 information [17]. At the same time, belief in conspiracy theories may predict fear of the coronavirus [18]. Consequently, there are numbers of the factors involved in the analysis and synthesis of information—whether it is reliable or inaccurate or completely false—about the coronavirus. In this context, epistemic motivation [19,20] and belief in conspiracy theories [21,22] may be significant predictors when it comes to the perception of coronavirus information and, as a consequence, may predict the fear of this infectious disease.

### 1.2. Conspiracy Theories

Conspiracy theories have been defined as beliefs that an individual or group has the ability to control the public and political order or part of it. Such conspiracy theories provide simple, understandable, and attractive explanations of complex phenomena. They can be also used as straightforward explanations for someone’s failure [22]. Conspiracy theories are also associated with beliefs that a group of individuals are working together and trying to achieve illegal and secret goals [23]. In this context, a belief in conspiracy theories could be thought to be associated with epistemic, existential, and social motives [21].

First, the epistemic motive is related to the desire for understanding, accuracy, and subjective certainty. It can be a plausible and effective explanation for new and unexpected events, where information is limited and advice is often inconsistent. Previous research suggests that a belief in conspiracy theories is associated with the need for cognitive closure, and particularly with respect to events that have no simple explanation [24]. Second, the existential motive is connected to a desire for control and security. This motive is also associated with a sense of security and control [25]. Third, the social motive is related to the desire to maintain a positive image of the self or a group.

Individuals who believe information from unreliable sources can isolate themselves from objective facts and institutions. There is often a lack of substantive evidence for conspiracy theories. However, they compensate for this by providing a framework for explaining complex phenomena in a simple way. Some research showed that conspiracy thinking may be negatively correlated with the level of analytical thinking and with education [23,26]. Moreover, a belief in conspiracy theories may be stronger when individuals experience anxiety resulting from uncertainty [27]. Conspiracy thinking can also be strongly related to a lack of social and political control, as well as psychological reinforcement [28]. Douglas et al. [21] showed that both conspiracy thinking and a belief in conspiracy theories result from the need for understanding, accuracy, and subjective certainty, but also the need to control and protect, as well as the desire to maintain a positive self-image within a group. According to Douglas’s theory [21] and previous studies, the need for cognitive closure could be an important factor related to conspiracy theories.

### 1.3. The Need for Cognitive Closure and Belief in COVID-19 Conspiracy Theories

The need for closure [19] is related to the search for and possession of clear, certain, and definitive knowledge to reduce the tension resulting from cognitive uncertainty. The intensity of this need determines the individual’s attitude to new data, as well as their method of processing the information [19,20]. The need for cognitive closure also affects the perception of the social world and the individual’s functioning in it [19,29]. A high level of the need for cognitive closure is associated with (1) shallow analysis of incoming information, and (2) motivation to search for information that is consistent with the individual’s existing structure of knowledge [19]. In addition, people with a high need for closure employ a superficial analysis of available information, and the information sought is aimed at confirming the adopted attitude or known stereotypes. In contrast, individuals with a low level of need for cognitive closure have a higher tolerance for ambiguity and uncertainty. They prefer a careful analysis of the data and are open to new information. As a result, they perceive the world in a complex, less stereotypical way, and seek alternative interpretations, assimilate new information, or adapt to changes [29].

Research has shown that belief in conspiracy theories can be predicted by the need for structure, the feeling of uncertainty, and a lower level of control [27]. In addition, an association between tolerance of ambiguity and beliefs about conspiracy theories has been found [30]. Moreover, there is some evidence that the need for predictability and closed-mindedness may predict beliefs in conspiracy theories [31]. In the context of COVID-19, Stoica and Umbreș [32] showed that open-mindedness, which was defined as the propensity to consider different points of view and the ability to change one’s mind and admit mistakes, was a predictor of a lower level of beliefs in conspiracy theories about the novel coronavirus. The authors postulated that epistemic humility may make people more likely to trust in expert knowledge that discredits false theories. Consequently, belief in COVID-19 conspiracy theories may be related to some dimensions of the need for cognitive closure, such as the need for predictability, avoidance of ambiguity, and closed-mindedness.

### 1.4. Belief in Conspiracy Theories about COVID-19 and Coronavirus Fear

As a result of restrictions introduced by local authorities during the pandemic, large numbers of people had to reduce the intensity of social activities they were able to attend or participate in. This may have contributed to the emergence of conspiracy theories and fake news regarding the origins and characteristics of the coronavirus [33]. Oleksy et al. [34] distinguished two types of coronavirus conspiracy theories: (1) general conspiracy theories related to a belief that the virus was created by individuals or groups who sought to profit from it, and (2) political conspiracies involving the belief that the authorities are withholding information about the actual causes of the pandemic outbreak. In this context, the perceived lack of individual control (feeling of powerlessness) was a predictor of the two types of conspiracy theories, while the sense of collective control correlated positively with general conspiracies and negatively with political conspiracy theories.

The relationship between belief in conspiracy theories and anxiety had already been observed before the pandemic outbreak [35]. Recent literature shows that conspiracy beliefs can also be regarded as a predictor of the perceived coronavirus threat [36]. Belief in conspiracy theories may result in an unrealistically low assessment of the risk from the new coronavirus and, thus, reduce fear [37], which may lead to a reduction in the incidence of health-promoting behaviors related to COVID-19 [38]. For example, people who believed in government conspiracies were less likely to use preventive measures, such as maintaining a social distance or hand washing [34]. Research in Latin America and Serbia showed that optimistic people who had a high level of trust and did not believe in conspiracy theories engaged more often in behaviors aimed at the prevention of coronavirus infection, and were less interested in stockpiling material goods during the pandemic [39]. Conversely, while pessimists with a low level of trust who believed in conspiracy theories also engaged in preventive behavior, they did tend to stockpile material goods and exhibited a higher level of anxiety. In addition, a negative correlation between a general belief in conspiracy theories and contact-related preventive behaviors was found [40]. However, while evidence suggests that conspiracy beliefs could predict fear of the coronavirus [18,37], findings regarding relationship between belief in conspiracy theories and health-protective behaviors vary between studies [18,40].

### 1.5. The Present Study

Previous research demonstrated a relationship between beliefs in COVID-19 conspiracy theories and the fear of the coronavirus [18]. A relationship between the need for cognitive closure and a belief in conspiracy theories was also found [30,31], as well as relationships between the need for cognitive closure and fear and anxiety [17]. Consequently, it can be surmised that the manner in which individuals analyze and search for coronavirus information is linked to both beliefs in COVID-19 conspiracy theories and coronavirus fear.

This assumption found support in empirical evidence. The results indicated that the need for cognitive closure was a predictor of anxiety and psychological distress [41,42] during the current pandemic [7]. Moreover, related psychological constructs, such as the need for structure, can be considered an important predictor of fear of the coronavirus [43]. Due to the unpredictability of the novel situation [44], people with a high level of intolerance to uncertainty may suffer increased psychological distress [45]. Moreover, a growing body of research shows that belief in conspiracy theories may result in underestimating the coronavirus threat [37,46,47]. It is possible that conspiracy beliefs lower the levels of anxiety and fear. Conspiracy theories, by providing a stable and understandable framework [22], can offer a false sense of security and familiarity with an uncertain situation. In particular, people with high levels of need for closure may reach for conspiracy theories, as they allow them to easily organize their perception of the world. Assessing coronavirus as not an actual threat, but as a lie or a manipulation, may reduce the level of anxiety caused by it.

By adopting this conceptual logic, we hypothesized that the relationship between the need for cognitive closure and the fear of the coronavirus is mediated by beliefs in COVID-19 conspiracy theories. In this context, studies suggested that three of the five dimensions of the need for closure may be of particular importance—the need for predictability, avoiding ambiguity, and closed-mindedness; these dimensions can be considered predictors of conspiracy beliefs [30,31]. For individuals with a high need for predictability, belief in conspiracy theories can reduce anxiety caused by the volatility and instability of situations. A similar relationship may take place with closed-mindedness. People with a high level of this trait may feel insecure because of the need to adapt to dynamic changes and reluctance to assume an alternative perspective. A simplified, schematic way of perceiving reality can, in turn, reduce psychological distress in people with a high level of avoidance of ambiguity. Therefore, we established the following detailed hypothesis: the relationship between the need for predictability (H1), avoidance of ambiguity (H2), closed-mindedness (H3), and coronavirus fear will be mediated by a belief in COVID-19 conspiracy theories.

Research to date suggests a relationship between fear of COVID-19 and age. The results of single studies [36,48], as well as meta-analyses [49], indicate a positive direction of this association. This effect can be explained by death anxiety [50]. The elderly are more susceptible to diseases and suffer more acutely from their effects [50,51]; hence, the risk of a novel disease should be associated with increasing fear. However, some studies provide contradictory results [52,53]. The negative direction of the correlation between these variables is explained by the fact that young people often live with family members in high-risk COVID-19 groups and, therefore, may intensify their fear for their loved ones [52].

There is also evidence of a link between age and belief in conspiracy theories. Although some results indicate a positive relationship [54], most studies show the opposite [38,46,55,56,57]. Some researchers suggest that younger people may be more likely to adopt such beliefs because of frequent use of social media, where a large amount of false information can be found [58], and because of less resistance to disinformation [59].

Given the evidence suggesting a relationship between these variables, as well as the evidence from previous studies [60], age was included in the model as a covariate of belief in conspiracy theories and fear of the coronavirus.

## 2. Materials and Methods

### 2.1. Participants

Four hundred thirteen participants (262 females) completed an online survey. The mean age of the participants was 33.70 years (SD = 11.90, age range: 15–70). To ensure the homogeneity of the sample, we focused on an adult cohort only. Therefore, the results for persons up to 20 years of age and over 60 years of age were excluded from the analyses (19 and 14 cases, respectively). The final sample consisted of 380 adults (239 females) with a mean age of 33.41 years (SD = 10.23). The study used snowball sampling. The link to the survey was shared on social media platforms and online forums with a request to pass the survey information on. The study was conducted between September 2020 and October 2020. During this period, the epidemic situation in Poland was characterized by the highest number of coronavirus cases, with an average of 4917 cases a day [61]. Lockdown and restrictions were introduced at that time, including an obligation to wear masks inside and outside public spaces and a limitation on the numbers of people allowed in public spaces [62]. Each participant in the survey was a volunteer and received no payment. The study was conducted in compliance with the Declaration of Helsinki. The participants were informed that their responses would be anonymous and confidential and that they could withdraw from the study at any time without giving any reason.

### 2.2. Measures

The Fear of COVID-19 Scale (FCV-19S) [6], which was adapted into Polish and validated (see the Supplementary Materials), comprises seven statements (e.g., “I cannot sleep because I’m worrying about getting coronavirus.”) rated by participants using a five-point Likert scale from 1 (strongly disagree) to 5 (strongly agree). Higher scores correspond to higher levels of coronavirus fear. The psychometric characteristics of the FCV-19S are presented in the Supplementary Materials.

The short version of the Need for Closure Scale [20,63], in a Polish adaptation by Kossowska, Hanusz, and Trejtowicz [64], was used to assess the need for cognitive closure dimensions. The scale consisted of 15 items. Individuals responded using a six-point scale from 1 (strong disagreement) to 6 (strong agreement). The questionnaire was composed of five subscales: (1) the need for order (Cronbach’s α = 0.78, McDonald’s ω = 0.79), (2) the need for predictability (Cronbach’s α = 0.76, McDonald’s ω = 0.76), (3) avoidance of ambiguity (Cronbach’s α = 0.56, McDonald’s ω = 0.56), (4) closed-mindedness (Cronbach’s α = 0.59, McDonald’s ω = 0.59), and (5) decisiveness (Cronbach’s α = 0.75, McDonald’s ω = 0.76). With brief scales, internal consistency coefficients may produce underestimated results [65,66]. Therefore, in this case, lower Cronbach’s α did not necessarily indicate low reliability. What was particularly important was that the α values were similar for those obtained in the Polish adaptation [64] and for those in the original version [20]. For these reasons, the overall reliability of the questionnaire was considered acceptable.

In order to measure beliefs on COVID-19 conspiracy theories S.S., J.M., and W.U. generated 57 items (20 facts, 18 opinions, and 19 conspiracy theories) about COVID-19. The majority of the factual information was obtained from the WHO webpages [67]. The selected information was related to popular facts about the coronavirus and the disease it causes. The opinions included items concerning how individuals are coping with the COVID-19 situation. The conspiracy theories were obtained by searching on social media and popular websites. Therefore, the items included conspiracy theories that were popular and had generated or were generating interest online at the time of our search. The items were evaluated by nine judges: eight psychology students (with four or five years of university education in psychology) and T.M. The judges rated each category (facts, opinions, and conspiracy theories) using a scale from 1 (the item does not fit the category, to 4 (the item fit the category). Moreover, each item was assessed by judges in terms of its acquaintance. The ten most valid items from each category were then selected. The details are presented in Table A1 in Appendix A. The respondents answered, on a 5-point Likert scale, to what extent they agreed with the presented information—the higher the score, the higher the level of belief in a fact, opinion, or conspiracy theory.

In the study, the items were ordered randomly. The higher the total score, calculated as the average of the component scores, the greater the level of the phenomenon. The Conspiracy Theories about the Coronavirus Scale that was prepared in this way showed excellent reliability, with Cronbach’s α = 0.93 and, McDonald’s ω = 0.94. The scale obtained an acceptable model fit in a confirmatory factor analysis: χ^2^ _(df = 30)_ = 89.56, *p* < 0.001, RMSEA = 0.072, 90% CI [0.055 0.090], CFI = 0.979, TLI = 0.969, and SRMR = 0.034. Considering modification indices [68], the covariates between items numbered 16 and 5, 25 and 17, 26 and 23, 20 and 24, and 21 and 23 were included.

### 2.3. Statistical Analysis

Pearson’s correlation coefficients were calculated to determine the relationships between the variables that were analyzed. To assess the relationship between the need for closure dimensions, coronavirus fear, age, and belief in conspiracy theories about the coronavirus, a structural equation analysis was conducted. We calculated the paths model using the maximum likelihood method with bootstrapping (5000 samples). Taking into account previous research [30,31], the model included the need for closure dimensions as predictors of the belief in conspiracy theories about the coronavirus. The need for closure dimensions and belief in conspiracy theories about the coronavirus were also considered as predictors of coronavirus fear [18]. The covariances between the need for closure dimensions, age with conspiracy beliefs, and age with coronavirus fear were included in the model. The following statistics were calculated as model-fit measures: χ^2^, RMSEA, SRMR, GFI, CFI, NFI, and TLI [68,69]. RMSEA and SRMR values lower than 0.08 may indicate that the model fit the dataset well [68]. In addition, GFI, CFI, NFI, and TLI values higher than 0.90 may indicate an acceptable fit of the model [68].

To verify the indirect effect between the need for closure dimensions and the coronavirus fear via the belief in conspiracy theories about the COVID-19, bootstrapping (5000 samples) with the bias-corrected percentile method was used [70,71]. For bootstrap procedures, the significance of the result is determined on the basis of the confidence interval. If zero is out of range, the result is considered possibly non-zero. When a significant indirect effect is detected, the association between the predictor and the dependent variable is assessed. If this relationship is no longer significant, mediation is defined as a full mediation. The case where the association between the predictor and the dependent variable remains significant is called the partial mediation [72]. It is also possible, initially, that there is no significant correlation between these variables, but the indirect effect turns out to be significant. This is called indirect-only mediation and is interpreted as full mediation [73].

The statistical calculations were conducted using the statistical software IBM SPSS (ver. 27.0.1.0) and R language’s [74] lavaan package (ver. 0.612) [75].

## 3. Results

Analysis showed that there was a negative correlation between coronavirus fear and belief in conspiracy theories about COVID-19. The coronavirus fear also correlated positively with avoidance of ambiguity and the need for predictability. However, a significant relationship between this variable, age, and closed-mindedness was not found. Belief in conspiracy theories about COVID-19 was positively associated with age. The detailed results are shown in Table 1.

**Figure 1:**
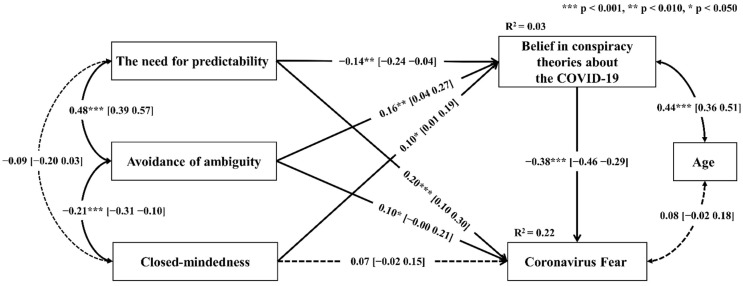
Paths model of relationships between the need for closure dimensions, belief in conspiracy theories about the COVID-19, age, and fear of the coronavirus (*n* = 380). The dashed line indicates statistically insignificant relationships.

The paths model showed an excellent fit with the data: χ^2^ _(df = 3)_ = 2.78, *p* = 0.426, RMSEA = 0.000, 90% CI [0.000 0.084], SRMR = 0.020, GFI = 0.998, CFI = 1.000, NFI = 0.991, and TLI = 1.004. The findings showed that the belief in conspiracy theories (β = −0.38, *p* < 0.001, 95% CI [−0.46 −0.29]), the need for predictability (β = 0.20, *p* < 0.001, 95% CI [0.10 0.30]), and avoidance of ambiguity (β = 0.10, *p* = 0.048, 95% CI [−0.00 0.21]) could be considered as the statistically significant predictors of coronavirus fear, with R^2^ = 0.22. Moreover, a significant path between belief in conspiracy theories and the need for predictability (β = −0.14, *p* = 0.007, 95% CI [−0.24 −0.04]), avoidance of ambiguity (β = 0.16, *p* = 0.002, 95% CI [0.04 0.27]), and closed-mindedness (β = 0.10, *p* = 0.033, 95% CI [0.01 0.19]) were found. Predictors explained 3% of variance of the dependent variable. Covariation between age and belief in conspiracy theories turned out to be statistically significant, with β = 0.44, *p* < 0.001, 95% CI [0.36 0.51]. On the other hand, no significant association between age and coronavirus fear was observed (β = 0.08, *p* = 0.090, 95% CI [−0.02 0.18]). The detailed results are presented in Figure 1.

Based on the bootstrapping procedures [70,71], significant indirect effects of belief in conspiracy theories about the COVID-19 were found with respect to the relationships between all dimensions of the need for closure and coronavirus fear. The results indicate a full mediation for avoidance of ambiguity and closed-mindedness and partial mediation for the need for predictability. The detailed results are shown in Table 2.

## 4. Discussion

The findings showed that beliefs in conspiracy theories about coronavirus may be considered as mediators of the relationship between the dimensions of the need for cognitive closure and the fear of coronavirus. The relationships with avoidance of ambiguity (H2) and closed-mindedness (H3) were fully mediated via belief in COVID-19 conspiracy theories. What is more, conspiracy beliefs partially mediated the relationship between preference for predictability and coronavirus fear (H1).

Avoidance of ambiguity was found to be positively related to an executive-thinking-style that characterized individuals who preferred to follow instructions rather than create their own [9,76]. People with high levels of need for closure tend to make less accurate decisions in uncertain or ambiguous situations [42]. Consequently, individuals with a high level of avoidance of ambiguity may be more likely to accept COVID-19 conspiracy beliefs without consideration. Conspiracy theories intensify in times of uncertainty and fear, providing simple explanations for new and incomprehensible events [22]. Therefore, recognizing the reality created by conspiracy theories eases psychological distress by simplifying an ambiguous, multi-dimensional situation.

A similar mechanism may be behind the relationship between coronavirus fear and closed-mindedness. Given the current findings, individuals with higher levels of closed-mindedness may refuse to accept, or ignore, information that contradicts their beliefs and, instead, rely on preconceptions that do not challenge their beliefs. As a result, they may be more resistant to new ideas and experiences and, thus, resistant to changes [77]. The direction of the relationship found in our study may be explained by the novelty of the situation. The rapid changes that took place over a short time may have made it difficult for people with high levels of closed-mindedness to adapt, increasing their insecurity and anxiety. In the context of COVID-19, those people may persist in their perception of the world situation and, as the result, more often adopt conspiratorial beliefs about the coronavirus. These beliefs, in turn, may lead to a reduction in the anxiety caused by the inconsistency of the current state with the previously adopted point of view on the factors that influence changes in the world.

In addition, a positive association between the need for predictability and the fear of the coronavirus was found. The situation in the initial periods of the pandemic changed dynamically, and the predictions about its course were ambiguous. Therefore, people with high levels of the need for predictability could lose the sense of control and ability to anticipate the situation. We observed a positive indirect effect between the need for predictability and coronavirus fear via beliefs in conspiracy theories. The direction of this relationship was not obvious and seemed counterintuitive. It is possible that conspiracy theories, while offering a coherent and unambiguous picture of the world, do not provide explanations that give a sufficient sense of stability. The motivations of people or organizations accused by conspiracy believers of being responsible for the current situation are not precisely known. Therefore, their further actions may be assessed as unpredictable.

The findings demonstrated a positive correlation between belief in conspiracy theories and age. Even though this relationship was observed previously [54], most of the earlier studies reported an association with an opposite direction, indicating that younger people are more likely to believe in conspiracy theories [38,46,55,56,57]. However, the literature provided inconsistent results. In some cases, no significant correlation between the variables was found [58,78,79]. Conspiracy theories offer a stable framework for understanding reality [22]. Because they explain novel events by referring to pre-existing elements (e.g., activities of recognizable people or organizations [2,3]), they may allow people to perceive the changing reality without having to revise the beliefs that have accompanied them for many years. Increasing belief in conspiracy theories may make it easier for older people to maintain a coherent and familiar vision of the world and to reduce anxiety about an unknown threat. Conversely, age demonstrated no significant correlation with the fear of the coronavirus. Work to date has produced conflicting results. Although some studies indicated the existence of such a relationship [36,48,52,53,80], there was also evidence suggesting no association between these variables [81,82,83]. There is also a discrepancy toward the direction of this association—some studies report a positive relationship [36,48,49], while others report a negative one [52,53]. Considering the vast diversity of the analyzed samples (e.g., Pakistani and Brazilian samples [53,54]), this discrepancy may result from intercultural differences. On the other hand, our sample consisted solely of adults aged 20 to 60 years. If the dynamic of severity of coronavirus fear is nonlinear, higher levels of this variable may occur only in certain (and in this case, extreme) age groups. The results of studies indicating intergroup differences in this aspect [83] supported this assumption. Because the belief in conspiracy theories is significantly correlated with both age and coronavirus fear, it is also possible that a more complex relationship may occur between these variables. Nevertheless, the relationship between these three variables has not been thoroughly investigated and requires further exploration.

A realistic assessment of a situation, especially one regarding potentially threatening issues, can increase the level of anxiety [37]. This phenomenon may be more intense in people who, due to a high level of need for cognitive closure, suffer more distress in novel and unpredictable situations. Belief in conspiracy theories can function as a defense mechanism, helping to deny the threat posed by the current situation. Denial of unpleasant facts about a threat has both negative and positive consequences. On the one hand, anxiety—especially related to the prospect of death—causes significant discomfort that the individual tries to reduce [84]. One technique for reducing tension may be to escape into the world of conspiracy theories, which provide a simple and, above all, less threatening explanation of complex situations. On the other hand, using such strategies may have serious consequences for the health of individuals and social groups, including disregarding the risk via less frequent use of preventive methods, such as maintaining social distancing [34]. What is more, a higher level of anxiety is associated with more frequent health-promoting behaviors, such as the use of personal protective equipment, more frequent hand washing, and the desire to be vaccinated [85,86].

The results of our research should be considered in light of this study’s limitations. First, snowball sampling, which may involve the selection of a specific group of respondents, was used. In addition, the survey was conducted online, as a result of which control over the research process was lower than it would have been with contact research. Another limitation was related to the form of our study. By using questionnaires, the results captured only the correlations between the variables, not the causal relationships. Therefore, the results should not be interpreted in a cause-and-effect manner. Finally, the relationship between the variables analyzed may vary from country to country, due to the broad diversity of actions taken by governments and the social trust in them [87].

## 5. Conclusions

Uncertainty and misinformation occurred during the COVID-19 pandemic, which may lead to the state of ambiguity [88]. This situation may increase anxiety or fear, especially in people with a high level of need for cognitive closure [7,41,42]. The dimensions of the need for cognitive closure, such as closed-mindedness, the need for predictability, and avoidance of ambiguity, are related to the processes of searching for and processing information [19,20]. The unpredictability of the situation and the presence of numerous items of negative information in the media [9] may increase psychological distress, especially in people with a high level of these characteristics. Belief in conspiracy theories allows them to restore or maintain a clear, orderly, and predictable vision of the world. Such beliefs provide a straightforward explanation for the outbreak of a pandemic by allowing threatening, unpleasant facts to be dismissed, which leads to reduced anxiety. Therefore, conspiracy beliefs may play a substantial role as the link between the manner in which individuals analyze and search for information and the fear of the coronavirus. Moreover, we found that age was positively correlated with belief in conspiracy theories, while demonstrating no significant correlation with fear of the coronavirus. Due to numerous conflicting results in previous studies, these relationships require further exploration, especially in a sample of older adults, who are most at risk of COVID-19.

## Figures and Tables

**Table 1:** Descriptive statistics and correlation between variables (*n* = 380).

Variables	*M*	*SD*	*s*	*k*	[1]	[2]	[3]	[4]	[5]
[1] Age	33.41	10.23	0.64	−0.70					
[2] Coronavirus fear	1.92	0.74	0.56	−0.52	−0.08				
[3] Belief in conspiracy theories	24.68	10.11	0.18	−1.05	0.42 ***	−0.38 ***			
[4] Avoidance of ambiguity	4.53	0.97	−0.55	−0.21	−0.01	0.16 **	0.08		
[5] The need for predictability	3.99	1.15	−0.29	−0.38	0.07	0.27 ***	−0.04	0.48 ***	
[7] Closed-mindedness	2.28	0.84	0.57	0.03	−0.04	0.00	0.06	−0.21 ***	−0.09

*Note*. *s*—skewness, *k*—kurtosis; *** *p* < 0.001, ** *p* < 0.01.

**Table 2:** Standardized indirect effects with bootstrap (5000 samples) 95% confidence intervals (*n* = 380) between need for closure dimensions and coronavirus fear via beliefs in conspiracy theories about coronavirus.

Model Pathways		Point Estimates	Standard Error	95% CI		*p*
Predictor	Mediator			Lower	Upper	
The need for predictability	Belief in conspiracy theories	0.05	0.02	0.01	0.09	0.009
Avoidance of ambiguity	Belief in conspiracy theories	−0.06	0.02	−0.11	−0.02	0.004
Closed-mindedness	Belief in conspiracy theories	−0.04	0.02	−0.08	−0.00	0.039

## Data Availability

All data are available from the corresponding author upon request.

## Supplementary Materials

The following supporting information can be downloaded at: https://www.mdpi.com/article/10.3390/ijerph192214789/s1, File S1: The Psychometric Properties of the Polish Version of the Fear of COVID-19 Scale.pdf [6,68,89,90,91,92,93,94,95,96,97,98,99,100,101,102,103].

## Appendix A

**Table A1:** The assessments of coronavirus facts, opinions, and conspiracy theories items from nine judges. The acquaintance of information contained in each item was also controlled.

Item	Evaluation	*M*	*SD*
^c^ The actions of the Polish authorities are effective in the fight against the coronavirus. [Działania polskich władz są skuteczne w walce z pandemią koronawirusa.]	Acquaintance	2.22	0.92
	Fact	2.11	0.99
	Opinion	3.44	1.07
	Conspiracy theory	1.67	1.05
^c^ There was no need to panic over the coronavirus. [Ludzie niepotrzebnie wpadli w panikę związaną z koronawirusem.]	Acquaintance	2.89	1.20
	Fact	2.33	0.94
	Opinion	3.44	0.96
	Conspiracy theory	1.44	0.50
^b^ Any prevention is advisable to avoid infection (disinfection, wearing masks). [Wszelka profilaktyka jest wskazana, aby uniknąć zarażenia (dezynfekcja, noszenie masek).]	Acquaintance	3.67	0.47
	Fact	3.67	0.47
	Opinion	2.56	1.34
	Conspiracy theory	1.11	0.31
^b^ People infected with the coronavirus but without symptoms can also spread the virus. [Osoby zarażone koronawirusem, ale bez objawów choroby, również mogą zarażać.]	Acquaintance	3.56	0.68
	Fact	3.67	0.67
	Opinion	1.67	1.05
	Conspiracy theory	1.67	1.05
^a^ The coronavirus was created by man. [Koronawirus został stworzony przez człowieka.]	Acquaintance	2.44	1.17
	Fact	1.78	0.79
	Opinion	2.56	1.26
	Conspiracy theory	3.56	0.96
^b^ Most people who get COVID-19 will recover. [Większość osób, które zachorują na COVID-19, wyzdrowieje z tej choroby.]	Acquaintance	3.33	0.67
	Fact	3.44	0.83
	Opinion	2.56	1.17
	Conspiracy theory	1.78	1.03
^b^ The coronavirus is a real threat to health and life. [Koronawirus stanowi rzeczywiste zagrożenie dla zdrowia i/lub życia.]	Acquaintance	3.33	1.05
	Fact	3.67	0.67
	Opinion	2.33	1.49
	Conspiracy theory	1.56	1.07
^b^ People of any age could be infected by the coronavirus. [Koronawirusem mogą zarazić się osoby w każdym wieku.]	Acquaintance	3.56	0.68
	Fact	3.89	0.31
	Opinion	2.00	1.15
	Conspiracy theory	1.67	1.05
^c^ People contract the coronavirus through their own inattention and failure to comply with restrictions. [Ludzie zarażają się przez własną nieuwagę oraz przez nieprzestrzeganie obostrzeń.]	Acquaintance	2.56	1.07
	Fact	2.67	1.15
	Opinion	3.33	1.05
	Conspiracy theory	1.56	0.96
^c^ Due to the media, the coronavirus seems to be more dangerous than it really is. [Przez media wydaje się, że koronawirus jest groźniejszy niż w rzeczywistości.]	Acquaintance	3.22	1.03
	Fact	2.78	1.03
	Opinion	3.56	0.68
	Conspiracy theory	2.22	1.23
^c^ General practicioners should not work remotely. [Lekarze rodzinni nie powinni pracować zdalnie.]	Acquaintance	2.56	1.17
	Fact	2.56	1.07
	Opinion	3.44	0.96
	Conspiracy theory	1.44	0.50
^b^ The coronavirus poses a greater threat to the elderly and those with comorbidities. [Koronawirus stanowi większe zagrożenie dla osób starszych oraz posiadających choroby współistniejące.]	Acquaintance	3.89	0.31
	Fact	3.89	0.31
	Opinion	2.22	1.31
	Conspiracy theory	1.44	0.68
^b^ Pneumonia vaccines do not protect against COVID-19. [Szczepionki przeciwko zapaleniu płuc nie chronią przed COVID-19.]	Acquaintance	1.67	1.05
	Fact	3.44	0.68
	Opinion	1.89	0.99
	Conspiracy theory	1.33	0.47
^a^ The obligation to wear face masks was introduced to control society. [Wprowadzono obowiązkowe noszenie masek na twarzy w celu kontroli społeczeństwa.]	Acquaintance	2.44	1.26
	Fact	1.89	1.29
	Opinion	2.67	1.33
	Conspiracy theory	3.78	0.42
^c^ Actions and restrictions introduced in the fight against the coronavirus were unnecessary. [Działania i obostrzenia wprowadzone w celu walki z koronawirusem były niepotrzebne.]	Acquaintance	3.22	0.92
	Fact	1.78	0.92
	Opinion	3.56	0.96
	Conspiracy theory	2.33	1.33
^a^ Chinese authorities have intentionally allowed the coronavirus to spread around the world. [Chińskie władze celowo dopuściły do rozprzestrzenienia się koronawirusa na świecie.]	Acquaintance	3.11	0.99
	Fact	1.22	0.42
	Opinion	2.78	1.31
	Conspiracy theory	3.78	0.42
^a^ The coronavirus tests results do not indicate its actual presence in the body (the results are random).] [Wyniki testów na obecność koronawirusa nie pokazują jego faktycznej obecności w organizmie (podane wyniki są losowe).]	Acquaintance	2.33	1.15
	Fact	1.56	0.68
	Opinion	3.11	0.99
	Conspiracy theory	3.33	1.05
^c^ Universities and other institutions should return to normal (pre-pandemic) operating mode. [Uczelnie wyższe oraz inne instytucje powinny wrócić do normalnego (takiego jak przed pandemią) trybu pracy.]	Acquaintance	3.89	0.31
	Fact	2.33	1.05
	Opinion	3.67	0.47
	Conspiracy theory	1.33	0.67
^c^ Wearing masks should be an individual decision. [Noszenie maseczek jest kwestią indywidualną.]	Acquaintance	2.56	0.96
	Fact	1.67	0.67
	Opinion	3.33	1.05
	Conspiracy theory	1.33	0.47
^a^ The coronavirus was created by the USA as a biological weapon. [Koronawirus został stworzony przez USA jako broń biologiczna.]	Acquaintance	2.44	1.26
	Fact	1.11	0.31
	Opinion	2.11	0.99
	Conspiracy theory	3.89	0.31
^a^ Coronavirus vaccines may contain microchips. [W szczepionkach przeciwko koronawirusowi mogą znajdować się mikroczipy.]	Acquaintance	2.33	0.94
	Fact	1.11	0.31
	Opinion	1.78	1.03
	Conspiracy theory	3.89	0.31
^c^ Schools should function as normal as before the pandemic. [Szkoły powinny funkcjonować normalnie—tak jak przed pandemią.]	Acquaintance	3.33	0.94
	Fact	1.89	0.99
	Opinion	3.44	0.96
	Conspiracy theory	1.33	0.47
^a^ 5G spreads the coronavirus. [Sieć 5G rozprzestrzenia koronowirusa.]	Acquaintance	2.56	1.17
	Fact	1.00	0.00
	Opinion	2.22	1.40
	Conspiracy theory	3.89	0.31
^a^ The coronavirus has become an element of the economic struggle against the dynamically developing Chinese economy. [Koronawirus stał się elementem walki ekonomicznej przeciwko prężnie rozwijającej się gospodarce Chin.]	Acquaintance	2.78	1.23
	Fact	1.56	0.68
	Opinion	2.56	1.26
	Conspiracy theory	3.44	0.83
^a^ The pandemic is political and media fraud. [Ogłoszona pandemia jest oszustwem medialno-politycznym.]	Acquaintance	3.78	0.63
	Fact	1.44	0.68
	Opinion	3.00	1.25
	Conspiracy theory	3.89	0.31
^a^ 5G has negative health effects or other negative consequences for society. [Sieć 5G negatywnie wpływa na zdrowie lub ma inne negatywne konsekwencje dla społeczeństwa.]	Acquaintance	2.56	1.07
	Fact	1.11	0.31
	Opinion	2.78	1.13
	Conspiracy theory	3.56	0.83
^b^ COVID-19 may be asymptomatic. [COVID-19 można przechodzić bezobjawowo.]	Acquaintance	3.89	0.31
	Fact	3.89	0.31
	Opinion	2.00	1.15
	Conspiracy theory	1.33	0.94
^b^ The virus can be transmitted airborne by sneezing and coughing, or by interacting with an infected person at a distance of less than one meter. [Wirus może być przenoszony drogą kropelkową poprzez kichanie i kaszel, bądź kiedy dochodzi do interakcji na odległość mniejszą niż 1 metr.]	Acquaintance	3.44	0.83
	Fact	3.89	0.31
	Opinion	2.11	1.20
	Conspiracy theory	1.33	0.47
^c^ Restrictions should be removed sooner. [Obostrzenia powinny zostać zniesione szybciej.]	Acquaintance	3.78	0.63
	Fact	2.00	1.25
	Opinion	3.56	0.68
	Conspiracy theory	1.56	0.96
^b^ The most common symptoms are fever, dry cough, and fatigue. [Najczęściej występującymi objawami są: gorączka, suchy kaszel, zmęczenie.]	Acquaintance	3.11	0.87
	Fact	3.44	0.68
	Opinion	1.89	1.10
	Conspiracy theory	1.44	0.68
Low temperature and snow cannot kill the coronavirus. [Niska temperatura i śnieg nie mogą zabić koronawirusa.]	Acquaintance	2.33	0.67
	Fact	2.67	1.15
	Opinion	3.00	0.94
	Conspiracy theory	1.78	1.03
In fact, COVID-19 is seasonal flu. [COVID-19 jest nową nazwą dla grypy sezonowej.]	Acquaintance	3.44	0.68
	Fact	1.78	0.79
	Opinion	2.67	0.94
	Conspiracy theory	2.33	1.25
The COVID-19 vaccine will contain a substance that causes infertility. [Szczepionka przeciw COVID-19 będzie w sobie zawierała substancję powodującą bezpłodność.]	Acquaintance	1.56	0.68
	Fact	1.11	0.31
	Opinion	2.44	1.34
	Conspiracy theory	3.78	0.42
Hand dryers are not effective against the coronavirus. [Suszarki do rąk nie są skuteczne w zabijaniu koronawirusa.]	Acquaintance	2.00	1.15
	Fact	3.33	0.82
	Opinion	2.44	1.07
	Conspiracy theory	1.22	0.63
COVID-19 was created to prevent Donald Trump from being re-elected. [COVID-19 został stworzony, aby Donald Trump nie został ponownie wybrany na stanowisko prezydenta USA.]	Acquaintance	1.89	0.99
	Fact	1.22	0.42
	Opinion	2.67	0.94
	Conspiracy theory	3.89	0.31
Pets are carriers of the coronavirus. [Zwierzęta domowe są nosicielami koronawirusa.]	Acquaintance	3.11	0.74
	Fact	1.78	1.03
	Opinion	2.11	0.99
	Conspiracy theory	2.89	1.29
The carrier of the coronavirus can infect others 2 days before the onset of the first symptoms. [Okres zarażania może zacząć się 2 dni przed pojawieniem się pierwszych symptomów.]	Acquaintance	2.33	1.25
	Fact	3.33	0.67
	Opinion	1.78	1.03
	Conspiracy theory	1.22	0.63
People suffering from allergy should not stop taking medications during the pandemic. [Alergicy nie powinni wstrzymywać się od przyjmowania leków podczas pandemii.]	Acquaintance	2.00	1.15
	Fact	3.44	0.96
	Opinion	2.00	0.94
	Conspiracy theory	1.44	0.96
Polish medical services are doing well in the fight against the coronavirus. [Polskie służby medyczne dobrze radzą sobie w walce z koronawirusem.]	Acquaintance	2.78	1.03
	Fact	2.56	0.96
	Opinion	3.22	0.79
	Conspiracy theory	1.22	0.42
There are no medications that can prevent or treat COVID-19. [Nie ma leków, które mogą zapobiegać lub leczyć COVID-19.]	Acquaintance	2.00	0.94
	Fact	2.11	1.20
	Opinion	2.56	1.17
	Conspiracy theory	2.44	1.17
Further isolation of the society is necessary to end the pandemic. [Dalsza izolacja społeczeństwa jest konieczna, aby udało się zakończyć pandemię.]	Acquaintance	2.89	0.87
	Fact	2.33	0.94
	Opinion	3.11	1.10
	Conspiracy theory	1.78	1.03
Hydrogen peroxide injections reduce the risk of coronavirus infection. [Wstrzykiwanie wody utlenionej zmniejsza ryzyko zakażenia koronawirusem.]	Acquaintance	1.33	0.67
	Fact	1.00	0.00
	Opinion	2.78	1.13
	Conspiracy theory	2.89	0.99
The coronavirus is artificial modification of a virus intended to cause panic. [Koronawirus to sztuczna modyfikacja wirusa stworzona w celu wywołania paniki.]	Acquaintance	2.22	1.03
	Fact	1.00	0.00
	Opinion	2.56	1.26
	Conspiracy theory	3.89	0.31
Bats are recognised as a natural reservoirs of the coronavirus. [Nietoperze są uznawane za naturalnych gospodarzy koronawirusa.]	Acquaintance	3.56	0.68
	Fact	3.33	0.82
	Opinion	2.11	1.10
	Conspiracy theory	1.56	0.68
Prolonged use of medical masks, if properly worn, will not result in CO_2_ poisoning or oxygen deficiency. [Długotrwałe stosowanie masek medycznych przy prawidłowym noszeniu nie powoduje zatrucia CO_2_ ani niedoboru tlenu.]	Acquaintance	2.22	1.03
	Fact	3.22	1.03
	Opinion	2.11	1.29
	Conspiracy theory	1.22	0.42
Quarantined persons should strictly follow the rules. [Osoby przebywające na kwarantannie powinny bezwzględnie jej przestrzegać.]	Acquaintance	3.67	0.67
	Fact	3.67	0.47
	Opinion	2.67	1.05
	Conspiracy theory	1.44	0.96
The restrictions are necessary to stop the spread of the coronavirus. [Wprowadzone obostrzenia są konieczne, aby koronawirus nie rozprzestrzeniał się.]	Acquaintance	3.56	0.50
	Fact	3.00	1.05
	Opinion	2.78	1.23
	Conspiracy theory	1.67	0.94
Fewer and fewer people are worried about the coronavirus situation. [Coraz mniej osób przejmuje się sytuacją związaną z koronawirusem.]	Acquaintance	3.56	0.68
	Fact	3.56	0.50
	Opinion	2.89	1.20
	Conspiracy theory	1.56	0.96
The coronavirus causes more deaths than seasonal flu. [Koronawirus powoduje więcej zgonów, niż grypa sezonowa.]	Acquaintance	3.22	0.92
	Fact	2.22	1.40
	Opinion	2.00	1.41
	Conspiracy theory	2.78	1.31
Being able to hold your breath for 10 s or more without coughing or feeling uncomfortable does not mean you are healthy and not infected with the coronavirus. [Możliwość wstrzymania oddechu przez 10 sekund lub dłużej bez kaszlu bądź poczucia dyskomfortu nie oznacza, że osoba jest zdrowa i nie jest zarażona koronawirusem.]	Acquaintance	2.11	1.20
	Fact	3.33	1.25
	Opinion	1.78	1.13
	Conspiracy theory	1.22	0.42
The virus was designed to control the size of the population. [Wirus ma na celu kontrolę liczebności ludności.]	Acquaintance	2.00	0.94
	Fact	1.44	0.68
	Opinion	2.22	1.23
	Conspiracy theory	3.78	0.42
Drinking alcohol might help remove the virus. [Spożywanie alkoholu może doprowadzić do pozbycia się wirusa.]	Acquaintance	1.67	0.67
	Fact	1.00	0.00
	Opinion	2.89	1.20
	Conspiracy theory	2.33	1.33
Media claims the coronavirus is a threat when, in fact, it is no more dangerous than seasonal flu. [Media określają koronawirusa jako zagrożenie, gdy w rzeczywistości nie jest on groźniejszy od grypy.]	Acquaintance	2.56	1.07
	Fact	2.33	0.94
	Opinion	3.56	0.96
	Conspiracy theory	2.56	1.07
Bill Gates has planned the pandemic. [Bill Gates zaplanował wprowadzenie stanu pandemii.]	Acquaintance	2.22	1.31
	Fact	1.44	0.68
	Opinion	2.33	1.05
	Conspiracy theory	3.89	0.31
SARS-CoV-2 is transmitted over the clouds. [SARS-CoV-2 przenoszony jest w chmurach.]	Acquaintance	1.11	0.31
	Fact	1.11	0.31
	Opinion	2.00	1.25
	Conspiracy theory	3.67	0.47
Following the development of a vaccine against the coronavirus, vaccination should be mandatory. [Gdy powstanie szczepionka przeciw koronawirusowi szczepienia powinny być obowiązkowe.]	Acquaintance	2.44	1.07
	Fact	2.56	1.17
	Opinion	3.00	1.25
	Conspiracy theory	1.89	1.29
The coronavirus can spread in hot and humid climates. [Koronawirus może rozprzestrzeniać się w gorącym i wilgotnym klimacie.]	Acquaintance	1.89	0.87
	Fact	2.78	1.13
	Opinion	2.00	1.05
	Conspiracy theory	1.78	1.03

*Note*. ^a^ item included in The Conspiracy Theories about the Coronavirus Scale; ^b, c^ item included as a buffer question (facts and opinions respectively); the original version in Polish is provided in square brackets.
