# Supplementary material for: The Relationship between the Need for Closure and Coronavirus Fear: The Mediating Effect of Beliefs in Conspiracy Theories about COVID-19
Source: Int J Environ Res Public Health. 2022 Nov 10;19(22):14789. doi: 10.3390/ijerph192214789 (PMC9690611; doi:10.3390/ijerph192214789)
Supplement: Supplementary file 1 [file ijerph-19-14789-s001.zip › File S1_The Psychometric Properties of the Polish Version of the Fear of COVID-19 Scale.pdf]

SUPPLEMENTARY MATERIALS

The Psychometric Properties of the Polish Version of the Fear of COVID-19 Scale

1. Materials and Methods

1.1. Participants

Two groups of participants completed an on-line questionnaire. The first group consisted of 431 individuals (273 females; *M* = 34.15 years; *SD* = 12.67; age range: 15-85). In this group, 16.2% of participants lived in a village, 13.5% lived in a small town (up to 20,000 residents), 21.8% lived in a medium town (20,000-99,000 residents), and 48.5% lived in a big town (over residents 100,000 residents). The second group consisted of 290 individuals (222 females; *M* = 27.62 years; *SD* = 8.46; age range: 15-59). In this group, 21.1% of participants lived in a village, 7.9% lived in a small town (up to 10,000 residents), 24.1% lived in a medium town (10,000-99,000 residents), and 46.9% lived in a big town (over residents 100,000 residents).

1.2. Methods

The Fear of COVID-19 Scale (FCV-19S) [1], which was adapted into Polish and validated, comprises seven statements (e.g., "I cannot sleep because I'm worrying about getting coronavirus.") rated by participants using a five–point Likert's scale from 1 – strongly disagree to 5 – strongly agree. Higher score correspond with higher level of coronavirus fear.

The Perceived Stress Scale (PSS 10) [2] in Polish adaptation by Juczyński [3] is a 10-item self-report scale assessing perceived stress. The answers are given on a four-point scale, from 0 (never) to 3 (very often). The higher the score reflect the greater severity of the perceived stress. The PSS 10 had good psychometric reliability with Cronbach's alpha equaled .90 in present study.

The Patient Health Questionnaire (PHQ-9) [4] in Polish adaptation by Kokoszka [5] is a 9-item self-report scale assessing depression symptoms. The answers are given on a four-point scale, from 0 (not at all) to 3 (nearly every day). The PHQ-9 had good psychometric reliability with Cronbach's alpha equaled .87 in present study.

Facebook use related to the coronavirus. The participants were to answer the questions about how often they used Facebook during the last month: 1) because friends placed information about the coronavirus; 2) to verify information about the coronavirus given in other media; and 3) to keep up to date with information about the coronavirus. They responded on a five-point scale from 1- never or almost never to 5-always or almost always.

1.3. Statistical Analysis

The analysis of the method structure was carried out on the first sample of participants (n = 431), whereas validation analysis was carried out on the second sample of participants (n = 290). The descriptive statistics like mean, standard deviation, skewness and kurtosis for all items and FCV-19S total score was calculated. Additionally, Pearson correlations coefficient between the items and between items and total score of FCV-19S was used. These statistics was calculated separately for first and second participant sample.

The first sample was randomly split into two subsamples before investigating the construct validity. More precisely, the factor structure of the FCV-19S was investigated by performing an EFA and CFA. Consequently, first subsample was used for EFA calculation (n = 217) and second subsample was used for CFA calculation (n = 214). For EFA, the Principal Axis Factoring extraction method was used. Additionally, the scree plot [6] and Kaiser [7] criterion was used to extract the number of factors. Moreover, the FCV-19S unidimensionality was verified using different coefficients such as mean of item residual absolute loadings (MIREAL), explained common variance (ECV), and unidimensional congruence [8]. Additionally, in order to confirm the single factor solution of FCV-19S, the CFA based on a maximum likelihood method was applied. The following statistics was used as assesses of model fit: χ^2, Root Mean Square Error of Approximation (RMSEA), Standardized Root Mean Square Residual (SRMR), comparative fit index (CFI), and Tucker-Lewis index (TLI). RMSEA lower than 0.08, SRMR lower than .08, and CFI and TLI values higher than .90 indicates a good fit of the model [9,10].

In order to assess the relationship between responses to particular item and the latent trait which the item is intended to assess, the item responses theory (IRT) analysis was used [11]. The seven items of the FCV-19S were analyzed using the graded response model (GRM) which enabled the analysis of ordinal response and rating scales [12]. In this model, the item discrimination parameter (α), range from $-\infty$ to $+\infty$, reflects the capability of the item to distinguish individuals at different levels of the latent trait. Additionally, threshold parameter (β) refers to the latent trait level in which the probability of answering above and below comparison response levels are equal. In this context, the theta (θ) reflects a unidimensional latent trait being assessed by the FCV-19S. The mean of theta (θ) equal 0 and the standard deviation of this latent trait equal 1. Additionally, the range of theta (θ) is arbitrary. It should be noted that, in GRM, each response level is compared to all response above that level. More specifically, there was four comparisons between levels because FCV-19S had five-point response scale. In this context, the first threshold (β1) described location on theta in first comparison when individuals were choosing to respond 1 versus all other responses. Analogically, the second threshold (β2) described location on theta in second comparison when individuals were choosing to respond 1 or 2 versus all other responses. Other thresholds such as β3 and β4 were described

analogously. It should be noted that items with negative α values and α values lower than 1 should be removed from the scale [11,13]. Additionally, in order to analyses the items of FCV-19S more accurately, the item response category characteristic curve (CCC) was conducted. The item response category characteristic curve presents the probability of individuals choose a certain response on the scale from 1 to 5 at various levels of the fear of coronavirus latent trait. The IRT analysis was carried out using data form the first sample of participants (n = 431).

The internal consistency of the FCV-19S was verified using following reliability coefficients: Cronbach's alpha, composite reliability, factor determinacy, and average variance extracted for the first sample of participants (n = 431). Moreover, the construct validity was assessed. More precisely, the relationship between coronavirus fear and perceived stress during last month, and between coronavirus fear and depression symptoms during last two weeks was calculated using Pearson correlations coefficient. Additionally, in order to construct validity analysis, the relationship between coronavirus fear and Facebook use related to the coronavirus was checked. The construct validity was carried out using data form the second sample of participants (n = 290).

The IBM SPSS Version 21 with AMOS 22 (descriptive statistics, criterion validation analysis), Stata 14 (CFA, IRT analysis) and Factor 10 (EFA, unidimensionality analysis) were using to conduct statistical analyses.

2. Results

2.1. Descriptives and Correlations

The descriptive statistics like mean, standard deviation, skewness and kurtosis for all items and FCV-19S total score were showed in Table S1. Additionally, there was a significant correlation between all items and between all items and FCV-19S total score. These descriptive statistics and correlation coefficients were presented separately for first (n = 431) and second (n = 290) sample.

Table S1. Descriptive statistics and correlation between seven items of FCV-19S in first and second sample.

Items	*M*	*SD*	Skewness	Kurtosis	Item 1	Item 2	Item 3	Item 4	Item 5	Item 6	Item 7
Sample 1 (*n* = 431)											
FCV-19S 1	3.46	1.24	-0.27	-1.18							
FCV-19S 2	3.43	1.31	-0.27	-1.30	.64						
FCV-19S 3	4.64	0.65	-2.26	6.77	.40	.37					
FCV-19S 4	3.89	1.24	-0.85	-0.50	.64	.55	.41				
FCV-19S 5	3.78	1.22	-0.65	-0.83	.42	.60	.38	.42			
FCV-19S 6	4.66	0.68	-2.34	6.25	.38	.36	.59	.48	.47		
FCV-19S 7	4.40	0.90	-1.61	2.18	.45	.44	.56	.49	.50	.69	
FCV-19S total score	4.04	0.78	-0.69	0.16	.79	.80	.64	.78	.74	.69	.75
Sample 2 (*n* = 290)											
Items	*M*	*SD*	Skewness	Kurtosis	Item 1	Item 2	Item 3	Item 4	Item 5	Item 6	Item 7
FCV-19S 1	2.82	1.24	0.08	-1.01							
FCV-19S 2	2.94	1.34	-0.02	-1.18	.59						
FCV-19S 3	1.26	0.62	2.77	8.54	.27	.29					
FCV-19S 4	2.00	1.22	1.05	0.00	.63	.44	.43				
FCV-19S 5	2.43	1.29	0.44	-0.99	.50	.60	.34	.46			
FCV-19S 6	1.31	0.72	2.94	9.77	.35	.33	.53	.43	.39		
FCV-19S 7	1.62	1.09	1.72	1.84	.47	.38	.52	.53	.41	.68	
FCV-19S total score	2.05	0.80	0.78	0.27	.79	.76	.58	.78	.76	.66	.75

Note. All correlation was statistical significance $p < .001$

2.2. Exploratory and Confirmatory Factor Analysis

The Kaiser–Meyer–Olkin Measure of Sampling Adequacy (KMO = .831) and Bartlett's Test of Sphericity ($\chi^2_{(21)} = 682.92$; $p < .001$) confirmed the appropriateness for conducting the EFA [14]. The EFA results showed that single factor explaining 55.56% of the total variance of the COVID-19 fear construct. Additionally, all factor loadings of FCV-19S items were higher than .50 threshold [15]. Consequently, all items formed a single factor (see Table S2). Additonally, the results of unidemensionality analysis showed that the MIREAL value equaled .27 which was below the

recommended threshold of .30. This result may indicated that unidimensional solution was no substantial bias. Additionally, the proportion of common variance attributable to the general factor (ECV) was .85, which was equal the threshold of .85. The unidimensional congruence equaled .98, which was above the desired threshold of .95. Taken together, these results may suggest that the structure of Polish version of the FCV-19S was unidimensional [8].

Table S2. The exploratory factor analysis results of the FCV-19S (n = 217).

Items	Factor loadings	95% Confidence Interval	
		Lower	Upper
1. I am most afraid of coronavirus.	.777	.709	.827
2. It makes me uncomfortable to think about coronavirus.	.772	.696	.817
3. My hands become clammy when I think about coronavirus.	.682	.575	.782
4. I am afraid of losing my life because of coronavirus.	.768	.693	.818
5. When watching news and stories about coronavirus-19 on social media, I become nervous or anxious.	.668	.560	.752
6. I cannot sleep because I'm worrying about getting coronavirus.	.763	.701	.825
7. My heart races or palpitates when I think about getting coronavirus.	.777	.675	.835
Eigenvalue			3.889
Total Variance Explained			55.56%

Based on the CFA, the one-factor structure of FCV-19S had unacceptable model fit: $\chi^2_{(df = 14)}$ = 155.05, p < .001, RMSEA = 0.217, SRMR = .080, CFI = .805, and TLI = .707. However, after adding the residual covariances between Items 1 and 2, Items 1 and 4, Items 2 and 4, Items 2 and 5, and Items 6 and 7, the model fit was acceptable: $\chi^2_{(df = 9)}$ = 21.18, p = .012, RMSEA = 0.080, SRMR = .033, CFI = .983, and TLI = .961. Factor loadings for all items ranged from .598 to .784 (see Table S3). Consequently, CFA and EFA results supported a one-dimensional factor structure of the Polish version of FCV-19S. Additionally, all items had an acceptable factor loadings level.

Table S3. The confirmatory factor analysis results of the FCV-19S (*n* = 214).

Items	Factor loadings	R - square
1. I am most afraid of coronavirus.	.598	.357
2. It makes me uncomfortable to think about coronavirus.	.599	.359
3. My hands become clammy when I think about coronavirus.	.714	.510
4. I am afraid of losing my life because of coronavirus.	.654	.428
5. When watching news and stories about coronavirus-19 on social media, I become nervous or anxious.	.688	.473
6. I cannot sleep because I'm worrying about getting coronavirus.	.722	.521
7. My heart races or palpitates when I think about getting coronavirus.	.784	.615

2.3. Item Responses Theory

The item threshold values (β) and item discrimination parameter (*α*) are presented in Table S4. In Figure 1, each curve in each plot represents the probability of choosing a certain response level. In this context, black curve (solid line) reflects change in the probability of selecting the first response level (strongly disagree) by individuals as a function of coronavirus fear latent trait. More specially, the probability of choosing this response level is high for individuals with low levels of coronavirus fear. However, this probability decreases as the level of coronavirus fear increases. At the same time, the probability of choosing the second response level (disagree) illustrated by black curve (dashed line) increases. Analogous changes of probability occur between consecutive response levels as coronavirus fear latent trait increases. Consequently, when the coronavirus fear is very high, individuals have a tendency to selecting the fifth response level (strongly agree). It should be noted that certain item is better at discriminating between individuals when the curves are peaked and dispersed across all of the latent trait [11,16].

Table S4. Item statistics for the graded response model (n = 431).

[3]

Items	α	β1	β2	β3	β_4
FCV-19S 1	2.51	-1.93	-0.71	-0.25	0.76
FCV-19S 2	2.43	-1.79	-0.60	-0.29	0.69
FCV-19S 3	2.57	-2.95	-2.77	-1.79	-0.73
FCV-19S 4	2.65	-1.87	-1.08	-0.68	0.10
FCV-19S 5	1.97	-2.38	-1.06	-0.65	0.35
FCV-19S 6	3.67	-2.84	-2.06	-1.54	-0.76
FCV-19S 7	3.49	-2.38	-1.64	-1.16	-0.38

2.4. Reliability Analysis

The results of internal consistency showed that the Cronbach's alpha equaled .86 and composite reliability equaled .87 which were above the recommended thresholds of .70 [17]. The factor determinacy was .99 which indicated high correlation between the factor score estimate and true factor score. Additionally, the factor determinacy was above the recommended thresholds of .80. Average variance extracted was .49 which was very close to the recommended threshold of .50. Taken together, these results may suggest that the Polish version of the FCV-19S presented good internal consistency [8].

2.5. Criterion-related Validity

The results of the construct validity showed that coronavirus fear assessed by FCV-19S was positively associated with perceived stress during last month and depression symptoms. during last two weeks. Additionally, there was a positively relationship between coronavirus fear and Facebook use related to the coronavirus. More precisely, the higher the coronavirus fear level, the more individuals used Facebook: 1) because friends place information about the coronavirus, 2) to verify information about the coronavirus given in other media, or 3) to keep up to date with information about the coronavirus. Detailed results are presented in Table S5.

Table S5. The relationship between coronavirus fear and additional variables.

Variables	*M*	*SD*	[1]	[2]	[3]	[4]	[5]
[1] Coronavirus fear	2.05	0.80					
[2] Perceived stress	2.11	0.82	.28***				
[3] Depression symptoms	1.25	0.71	.21***	.74***			
How often do you use Facebook during the last month:							
[4] because friends place information about the coronavirus	1.27	0.69	.21***	.21**	.17**		
[5] to verify information about the coronavirus given in other media	1.63	1.03	.20**	.21***	.13*	.64***	
[6] to keep up to date with information about the coronavirus	1.63	1.02	.24***	.18**	.14*	.65***	.84***

Note. *** p < .001, ** p < .01, * p < .05

References

1. Ahorsu, D. K.; Lin, C. Y.; Imani, V.; Saffari, M.; Griffiths, M. D.; Pakpour, A. H. The fear of COVID-19 scale: development and initial validation. *Int J Ment Health Addict* **2020**, *20*, 1537-1545. https://doi.org/10.1007/s11469-020-00270-8.
2. Cohen, S.; Kamarck, T.; Mermelstein, R. A global measure of perceived stress. *J Health Soc Behav* **1983**, *24*, 385-396. https://doi.org/10.2307/2136404.
3. Juczyński, Z. *Narzędzia pomiaru w promocji i psychologii zdrowia*; Pracownia Testów Psychologicznych Polskiego Towarzystwa Psychologicznego**:** Warszawa, Poland, 2001.
4. Kroenke, K.; Spitzer, R. L.; Williams, J. B. The PHQ-9: validity of a brief depression severity measure. *J. Gen. Intern. Med.* **2001**, *16*, 606-613. https://doi.org/10.1046/j.1525-1497.2001.016009606.x.
5. Kokoszka, A.; Jastrzębski, A.; Obrębski, M. Ocena psychometrycznych właściwości polskiej wersji Kwestionariusza Zdrowia Pacjenta-9 dla osób dorosłych. *Psychiatria* **2016**, *13*, 187-193.
6. Cattell, R. B. The scree test for the number of factors. *Multivar. Behav. Res.* **1966**, *1*, 245–276. http://dx.doi.org/10.1207/s15327906mbr0102_10.
7. Kaiser, H. F. The application of electronic computers to factor analysis. *Educ Psychol Meas* **1960,** *20*, 141–151. http://dx.doi.org/10.1177/001316446002000116.
8. Ferrando, P. J.; Lorenzo-Seva, U. Assessing the quality and appropriateness of factor solutions and factor score estimates in exploratory item factor analysis. *Educ Psychol Meas* **2017**, *78,* 762-780. https://doi.org/10.1177/0013164417719308.
9. Hu, L. T.; Bentler, P. M. Cutoff criteria for fit indexes in covariance structure analysis: Conventional criteria versus new alternatives. *Struct. Equ. Model.* **1999**, *6*, 1–55. https://doi.org/10.1080/10705519909540118.
10. Kline, R. B. *Principles and practice of structural equation modeling*; Guilford Press: New York, USA, 2011.
11. Hambleton, R. K.; Swaminathan, H. *Item response theory: Principles and applications*; Springer: New Jersey, USA, 2013.
12. Samejima, F. Graded Response Model. In *Handbook of Modern Item Response Theory;* van der Linden, W. J., Hambleton, R. K., Eds.; Springer: New Jersey, USA, 1997.
13. Yang, F. M.; Kao, S. T. Item response theory for measurement validity. *Shanghai Arch. Psychiatry* **2014**, *26*, 171-177. https://doi.org/10.3969/j.issn.1002-0829.2014.03.010.
14. Hair, J. F.; Black, W. C.; Babin, B. J.; Anderson, R. E. *Multivariate data analysis. A global perspective*; Pearson Education: New York, USA, 2010.
15. Ferguson, E.; Cox, T. Exploratory factor analysis: A users' guide. *Int. J. Sel. Assess.* **1993**, *1*, 84–94. http://dx.doi.org/10.1111/j.1468-2389.1993.tb00092.x.
16. Embretson, S. E.; Reise, S. P. *Item response theory for psychologists*; Lawrence Erlbaum Associates: Mahwah, USA, 2000.
17. Nunnally, J. C.; Bernstein, I. H. *Psychometric theory,* 3rd ed; McGraw-Hill: New York, USA, 1994.
